# Multiplexed electrospraying of water in cone-jet mode using a UV-embossed pyramidal micronozzle film

**DOI:** 10.1038/s41378-022-00391-1

**Published:** 2022-09-29

**Authors:** Ji-hun Jeong, Kwangseok Park, Hyoungsoo Kim, Inyong Park, Jinyoung Choi, Seung S. Lee

**Affiliations:** 1 Department of Mechanical Engineering, Korea Advanced Institute of Science and Technology, Daejeon, 34141 Republic of Korea; 2 Department of Environmental Machinery, Korea Institute of Machinery and Materials, Daejeon, 34103 Republic of Korea; 3 Department of Mechanical Engineering, Dongshin University, Naju, 58245 Republic of Korea

**Keywords:** Nanofluidics, NEMS

## Abstract

The electrospraying of water in the cone-jet mode is difficult in practical applications owing to its low throughput and the electrical discharge caused by the high surface tension of water. A film with multiple dielectric micronozzles is essential for multiplexed electrospraying of water in cone-jet mode without electrical discharge. Thus, a pyramidal micronozzle film with five nozzles was fabricated using the UV-embossing process. The pyramidal micronozzle film consisted of pyramidal micronozzles, a micropillar array, and an in-plane extractor, which were proposed to minimize wetting and concentrate the electric field to the water meniscus at the tip of the pyramidal micronozzle. The electrospraying of water using a single pyramidal micronozzle was visualized by a high-speed camera at a flow rate of 0.15–0.50 ml/h with voltages of 0.0–2.3 kV, −1.6 kV, and −4.0 kV at the water, guide ring, and collector, respectively. Three distinct modes, the dripping, spindle, and cone-jet modes, were observed and distinguished according to the motion of the water meniscus at the nozzle tip. The steady Taylor cone and jet were observed in a voltage range of 1.3–2.0 kV in water, particularly in cone-jet mode. Multiplexed electrospraying of water in cone-jet mode at a flow rate of 1.5 ml/h was performed using a pyramidal micronozzle film, demonstrating the potential for a high-throughput electrospraying system.

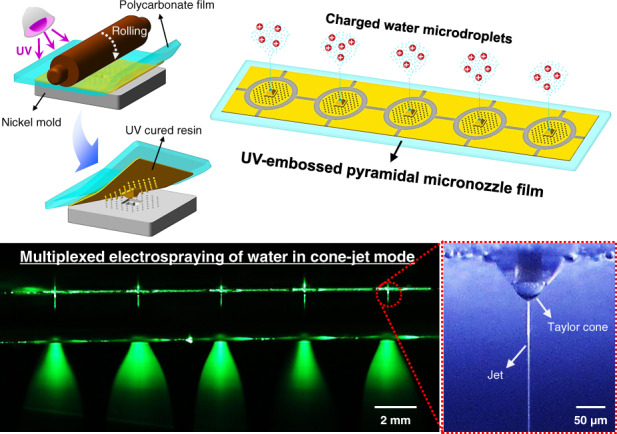

## Introduction

**Fig. 1:**
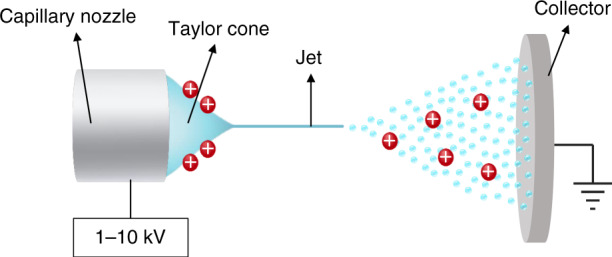
Electrospraying using a capillary nozzle-collector configuration. Electrospraying is typically conducted by using a capillary nozzle-collector configuration. A thin jet is emitted from the apex of the Taylor cone and disintegrated into charged microdroplets. The droplets have identical polarities and accumulate on the grounded collector

Electrospraying is a spray method that can generate fine, charged droplets^1,2^. Electrospraying has many applications, such as patterning^3–5^, mass spectrometry^6,7^, drug delivery^8,9^, and micro combustion^10,11^. In a general electrospray setup, a capillary nozzle with a high electric potential of 1–10 kV is placed perpendicular to a grounded collector (Fig. 1). The electric field at the nozzle drives the liquid into a conical shape known as a Taylor cone and causes the emission of a thin jet at the apex of the cone. The jet breaks into fine, charged droplets, and the droplets do not coalesce owing to the electrical repulsion between particles of the same polarities. When the electric potential reaches the threshold, a steady emission of the jet and droplets occurs; this emission is referred to as the cone-jet mode^2,12^. In cone-jet mode, charged, monodisperse micro/nanodroplets are continuously produced, making this mode distinguishable from other electrospray modes.

Although electrospraying is a widely used method, electrospraying of water is still considered a challenging task owing to the high surface tension of water. Several studies have shown that the electrospraying of water in ambient air without electrical discharge is impossible using a metallic capillary nozzle^13,14^. The ionization of air occurs before the cone-jet mode reaches the onset voltage, resulting in unstable Taylor cone formation and the generation of harmful gases such as ozone. Using a small dielectric capillary nozzle with deionized water is reported to be an appropriate solution for stabilizing the electrospraying of water^15–17^.

The low-throughput characteristic of electrospraying in cone-jet mode is a severe drawback for many applications. It has been reported that the narrow and low flow rate condition (0.1–0.6 ml/h) is also indispensable for the electrospraying of water by a small capillary nozzle to maintain cone-jet mode without electrical discharge^15,16^, whereas most applications, such as fine dust collection^18,19^, antimicrobial processes^20,21^, deodorization^22^, crop spraying^23^, and desalination of water^24^, require high-throughput. These applications are suitable for electrospraying because of the electrical charge and reactive oxygen species inside the water microdroplets. A recent study reported that a cylindrical polymer micronozzle can also perform electrospraying of water in cone-jet mode without electrical discharge, and this method has the potential for development into a multiplexed electrospraying system^25^. However, the extension of its applicability is still limited owing to the lack of productivity in micronozzle fabrication.

In this study, multiplexed electrospraying of water was performed using a pyramidal micronozzle film. The proposed pyramidal micronozzle film was fabricated using the UV-embossing process. This film stands out in terms of the fabrication speed and cost compared to typical electrospray nozzles such as the silicon micronozzles fabricated by the deep reactive ion etching process^11,26,27^ and the metallic micronozzles fabricated by CNC machining^28^. The pyramidal micronozzle provided suitable conditions for embossing and localizing the water meniscus at the tip of the nozzle to concentrate the electric field. A micropillar array prevented surface wetting during the electrospraying of water. The stability of the electrospray based on the flow rate and voltage was evaluated by high-speed imaging using a single pyramidal micronozzle. An experiment with a linear array of five pyramidal micronozzles in a film was performed to demonstrate the multiplexed electrospraying of water in cone-jet mode.

## Results and discussion

### Design and fabrication of the pyramidal micronozzle film

**Fig. 2:**
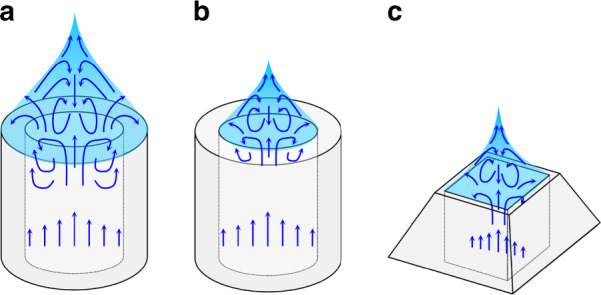
Schematic diagram of the Taylor cone formation based on the nozzle tip design. **a** Hydrophilic tip. The flow between the outer and inner rims of the nozzle leads to the unstable form of the Taylor cone. **b** Hydrophobic tip. The flow between the outer and inner rims of the nozzle is not created due to the hydrophobic surface. **c** Pyramidal tip. The gap between the outer and inner rims is geometrically reduced. The effects of flow on the formation of the Taylor cone can be minimized even if wetting occurs at the tip

Figure 2 shows the Taylor cone formation based on the nozzle tip design. A Taylor cone is formed based on the outer rim of the nozzle when the tip is hydrophilic (Fig. 2a). A flow is created to fill the space between the outer and inner rim of the nozzle, resulting in the unstable flow of the Taylor cone^25,29^. A hydrophobic tip can anchor the base of the Taylor cone to the inner rim of the nozzle (Fig. 2b). This approach is effective for creating a steady cone-jet mode^25,29–31^. However, excessive flow or an imperfect hydrophobic surface coating may cause the nozzle tip to become wet. A pyramidal tip with a geometrically reduced outer rim could anchor the base of the Taylor cone to the inner rim of the nozzle (Fig. 2c). Even if the base of the Taylor cone exceeds the inner rim owing to the imperfect hydrophobic coating at the tip, a steady cone-jet mode is expected to be achievable because the gap between the outer and inner rims is small.

**Fig. 3:**
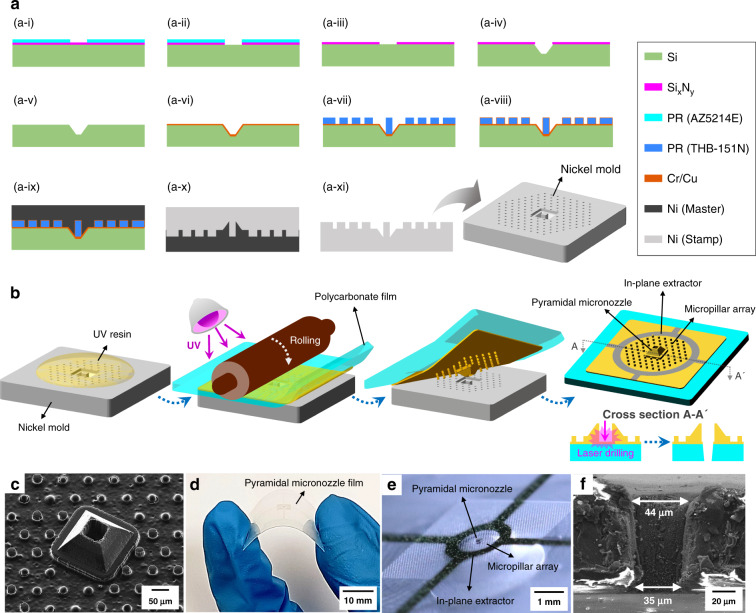
Fabrication process and results of the pyramidal micronozzle film. **a** Fabrication of the nickel mold. Bulk micromachining and photolithography on a single crystalline silicon substrate were conducted to create an opposite pattern to that of the pyramidal micronozzle and the micropillar array (**a-i** to **a-viii**). The patterned substrate was replicated by electroforming nickel (**a-ix** to **a-xi**). **b** Fabrication process of the pyramidal micronozzle film. The pyramidal micronozzle and micropillar array was fabricated on a polycarbonate film via the UV-embossing process. Multiple layers of chromium and copper were deposited to pattern the in-plane extractor. Laser drilling was conducted to form a hole at the nozzle. The pyramidal micronozzle film was dip-coated in HDFS solution to create a hydrophobic surface. **c** SEM image of the UV-embossed pyramidal micronozzle and the micropillar array. **d** The pyramidal micronozzle film after removal from the nickel mold. The fabricated film was flexible. **e** Deposition results of the in-plane extractor. **f** Laser drilling results of the polycarbonate film

Figure 3a shows the process of fabricating a nickel mold. An AZ5214E photoresist layer with a thickness of 2 μm was spin-coated and patterned on a 300-nm thick silicon nitride layer deposited on a silicon substrate (Fig. 3a-i). The exposed silicon nitride was etched using a reactive ion etching process (Fig. 3a-ii). The residual AZ5214E photoresist was removed using acetone to reveal the silicon nitride mask pattern for silicon bulk etching (Fig. 3a-iii). Silicon was bulk-etched with a 45 wt% potassium hydroxide solution at 85 °C (Fig. 3a-iv). Mold cavities with a depth of 62 μm were formed for the pyramidal micronozzle using this process. The remaining silicon nitride was removed using a reactive ion etching process (Fig. 3a-v). A chromium layer of 30 nm and a copper layer of 100 nm were deposited on the bulk micromachined silicon substrate (Fig. 3a-vi). A THB-151N photoresist layer with a thickness of 30 μm was spin-coated and patterned on the substrate (Fig. 3a-vii). Mold cavities for the holes of the pyramidal micronozzles and the micropillar array are formed by this process. An additional chromium layer of 30 nm and a copper layer of 100 nm were deposited on the patterned THB-151N substrate (Fig. 3a-viii). A nickel master mold was electroformed to a thickness of 300 μm and detached from the bulk-etched silicon substrate (Fig. 3a-ix). The residual chromium, copper and THB-151N in the nickel master mold were removed. A nickel stamp mold, which was used as the mold for the UV-embossing process, was electroformed to a thickness of 200 μm (Fig. 3a-x, xi). Figure 3b shows the fabrication of the pyramidal micronozzle film. UV-curable polyurethane acrylate resin (M5027, UVIS material technology, Republic of Korea) was dispensed onto the nickel mold. The UV resin was attached to a 100-μm-thick polycarbonate film and filled into the nickel mold by applying pressure during rolling. The UV resin was then cured for 30 s using a mercury UV lamp (100 mW/cm^2^). The pyramidal micronozzle and the micropillar array were transferred by peeling the polycarbonate film. Figure 3c shows the SEM image of the UV-embossed pyramidal micronozzle and the micropillar array. The inner and outer rims were 60 μm and 88 μm in size, respectively, and the height of the tip was 92 μm. The micropillar array had a pillar diameter of 25 μm and pillar height of 30 μm and was arranged in a triangular pattern with a spacing of 55 μm. The flexible pyramidal micronozzle film was produced rapidly, and a film with multiple pyramidal micronozzles was easily produced (Fig. 3d). E-beam evaporation was applied to deposit the in-plane extractor with multilayers of 30, 150, and 50 nm of chromium, copper, and chromium, respectively (Fig. 3e). A metal shadow mask with an in-plane extractor pattern of 1.2 mm diameter and 300-μm line width was aligned with the pyramidal micronozzle before deposition. A circular hole was drilled into the polycarbonate film using a femtosecond laser machining system. The hole sizes were measured to be 44 and 35 μm at the top and bottom, respectively (Fig. 3f). The pyramidal micronozzle film was attached to a rigid polycarbonate substrate using different UV resins (USM-520D, UVIS material technology, Republic of Korea). A fluorocarbon solution of HDFS (heptadecafluoro-1,1,2,2-tetrahydrodecyltrichlorosilane) diluted with n-hexane in a volume ratio of 1:1000 was additionally dip-coated onto the pyramidal micronozzle film to increase the hydrophobicity of the nozzle and the micropillar array^25,32^. The pyramidal micronozzle film was dried for 60 min in ambient air after 5 min of dip-coating. The water meniscus adhered to the inner rim of the nozzle during the pendant drop test (Supplementary Fig. S1). The static contact angle of the micropillar array was 134° in the Cassie–Baxter state.

### Single electrospraying of water in cone-jet mode

**Fig. 4:**
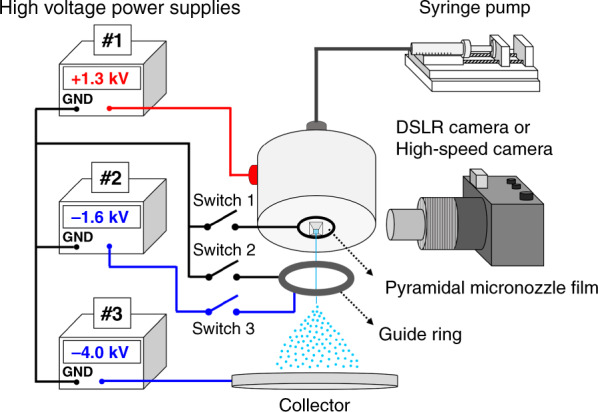
Experimental setup for electrospray visualization. The main high voltage was applied to the water. The in-plane extractor was connected to switch 1 to identify the effect of electric concentration at the nozzle tip. A guide ring was connected to switches 2 and 3. Switches 2 and 3 were not connected simultaneously. Switch 2 was connected when switch 1 was disconnected. Switch 3 was connected when switch 1 was connected (the grounded in-plane extractor). The flow rate was controlled using a syringe pump. A DSLR camera or a high-speed camera was used for the visualization of the electrospray

A schematic of the experimental setup for the high-speed visualization of the electrospray of water is shown in Fig. 4. The surface tension and electrical conductivity of water were measured as 0.072 N/m and 1.4 × 10^−4^ S/m, respectively. The electrospray was operated using a syringe pump at three high-voltage powers. A voltage of up to 2.3 kV was applied to the water. A guide ring with a 6 mm hole and a collector with a diameter of 100 mm were placed 2 mm and 20 mm away from the pyramidal micronozzle film. Voltages of −1.6 and −4.0 kV were applied to the guide ring and collector, respectively, to induce the charged water microdroplets toward the collector. Three switches were used to verify the effect of the in-plane extractor. Switch 2 was connected when the in-plane extractor was not in use, whereas switches 1 and 3 were connected when the in-plane extractor was in use. A DSLR camera equipped with a macro lens and an objective lens (×10 magnification) was used to visualize the electrospray. A high-speed camera was used to capture images of the short moments in the individual electrospray modes. The electrospray of water was operated at a flow rate of 0.15–0.50 ml/h.

**Fig. 5:**
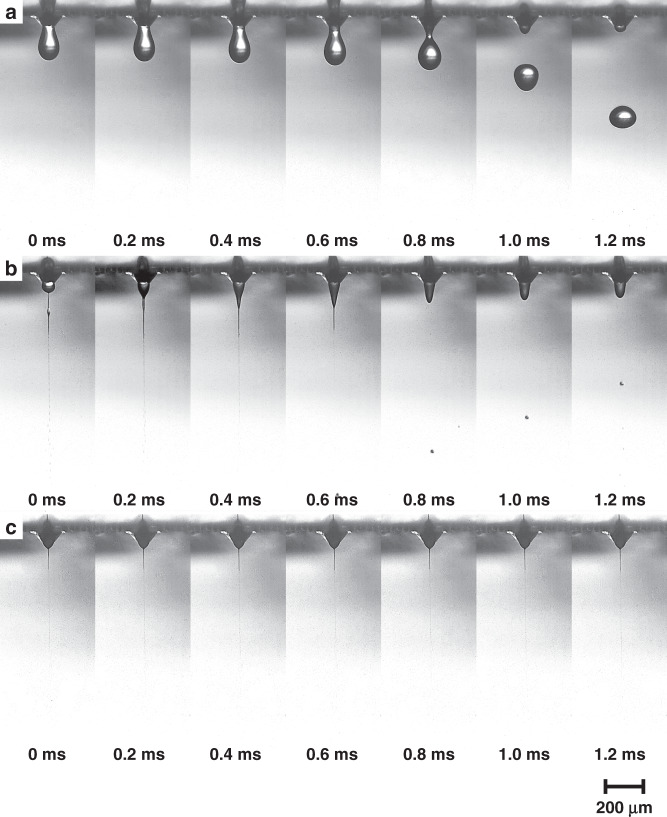
High-speed images of the electrospraying of water by the pyramidal micronozzle at a flow rate of 0.15 ml/h. **a** Dripping mode (0.8 kV). **b** Spindle mode (1.1 kV). **c** Cone-jet mode (1.3 kV)

Figure 5 shows high-speed images of the electrospray modes at a flow rate of 0.15 ml/h. The images were recorded at a time interval of 0.2 ms (5000 fps) and an exposure time of 260 ns. Three distinct modes were observed depending on the voltage applied to the water: dripping, spindle, and cone-jet modes. In the dripping mode, pulsation of the nozzle-sized microdroplets was observed in the low voltage range of 0.6–0.9 kV (Fig. 5a). The period of the dripping and size of the microdroplet decreased as the voltage was increased. The spindle mode was observed in the voltage range of 0.9–1.2 kV. A blunt water meniscus formed, and the jet was intermittently emitted from the apex of the meniscus (Fig. 5b). The nonuniformly sized water microdroplets disintegrated from the jet. A steady formation of the Taylor cone and jet was observed in cone-jet mode, which was operated in the voltage range of 1.3–1.8 kV (Fig. 5c). Unlike the previous modes, the emission of the jet was continuous, and uniform-sized microdroplets were disintegrated from the jet. In addition, an intermittent jet was observed at a flow rate of 0.10 ml/h, which indicates that the observation agrees with the minimum flow rate of 0.147 ml/h calculated from the theory for polar liquid^33,34^.

**Fig. 6:**
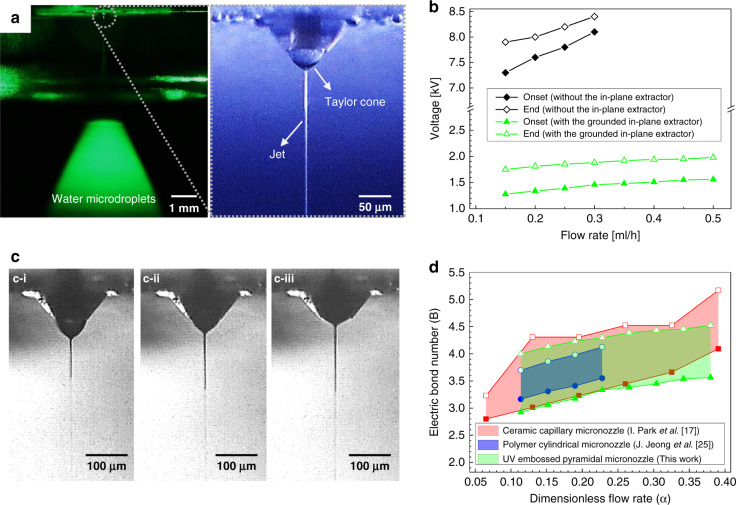
Experimental results of the cone-jet mode. **a** DSLR camera images in cone-jet mode (voltage at water: 1.6 kV, flow rate: 0.50 ml/h). **b** Onset and end voltages of the cone-jet mode. The data plotted in black lines with diamond symbols are the results when switch 2 depicted in Fig. 4 was connected. The data plotted in green lines with triangle symbols are the results when switches 1 and 3 depicted in Fig. 4 were connected. **c** Morphology of the Taylor cone and jet at voltages of 1.3 kV (**c-i**), 1.5 kV (**c-ii)**, and 1.7 kV (**c-iii**) at a flow rate of 0.15 ml/h. The meniscus of the Taylor cone was compressed toward the nozzle as the applied voltage increased. **d** Operation domain of the steady cone-jet mode of water. The domains of the other research studies were replotted from the data provided in the references. The domain obtained using UV-embossed pyramidal micronozzles showed good agreement with the other domains obtained by the other types of micronozzles

Figure 6 shows the detailed experimental results of the cone-jet mode. The water microdroplets were visualized using a laser sheet perpendicular to the in-plane extractor and the view direction of the camera (Fig. 6a). Figure 6b shows the onset and end voltages of the cone-jet mode with respect to the flow rate. The flow rate limit for the steady cone-jet mode was found to be 0.30 ml/h when the in-plane extractor was not used. This result is based on the observation of the electrical discharge caused by excessive flow at the nozzle tip when the flow rate was 0.35 ml/h without the use of the in-plane extractor. However, a flow rate of up to 0.50 ml/h was possible when the in-plane extractor was grounded. This result indicates that localizing the electric field by the in-plane extractor also affects the operating range of the flow rate. The operating voltage at the water interface was reduced by nearly six times when the in-plane extractor was grounded, which is similar to the result of a previous study^25^. The gap between the onset and end voltages was ~0.4–0.6 kV, which is slightly larger than that in a previous study^25^. The electrical discharge occurred when the applied flow rate or the applied voltage exceeded the limits. These results indicate that the onset of the electrical discharge can be delayed through the concentrated electric field of the in-plane extractor, which can increase the operating flow rate range and lower the operating voltage. Figure 6c-i–c-iii shows the change in the Taylor cone shape with respect to the voltage at a flow rate of 0.15 ml/h. The Taylor cone was deformed toward the nozzle since a high degree of electric stress was applied to the water meniscus owing to the increase in the voltage. The jet was not observed under a condition above 2.0 kV. Corona discharge occurred near the tip of the pyramidal micronozzle when the voltage was increased to 2.3 kV. The surface of the pyramidal micronozzle became hydrophilic after discharge. However, the electrospraying of water in cone-jet mode was still operational due to the tapered geometry of the nozzle, which minimized the severe wetting of the nozzle (Supplementary Fig. S2). The jet diameter increased from 3.4 to 3.8 μm, and further investigation on the effect of the jet diameter on meniscus size is required, as this topic has not been discussed in classical electrospray theory. The operation domain of the electrospraying of water in cone-jet mode was obtained based on high-speed image observations (Fig. 6d). Two parameters, the dimensionless flow rate (*α*) and electric bond number (*B*), were used to represent the operation domain.

$$\alpha = \frac{{\rho KQ}}{{\varepsilon _0\kappa \gamma }},\;B = \frac{{4V}}{{\ln \frac{{8H}}{d}}}\sqrt {\frac{{\varepsilon _0}}{{\gamma d}}}$$

*ρ* is the density of water, *K* is the electrical conductivity of water, *Q* is the flow rate, *ε*_0_ is the vacuum permittivity, *κ* is the relative permittivity of water, *γ* is the surface tension of water, *V* is the voltage applied to water, *H* is the distance between the nozzle tip and the grounded electrode, and *d* is the inner diameter of the nozzle. The derivation of the electric bond number was based on the electric field at the nozzle in a tip-to-plane configuration, as suggested in previous studies^35,36^. The height of the pyramidal micronozzle tip was used as the distance between the nozzle tip and grounded electrode because the in-plane extractor was considered to be the grounded electrode. The electric field induced from the other voltage sources (the guide ring and collector) was neglected in the calculation of the electric bond number because the electric field concentration initiated by the in-plane extractor was dominant^25^. Although the electric bond number was simplified, the operation domain of the steady cone-jet mode almost matched the domain represented in a previous study^17^.

Unlike previous studies in which pulsation of the Taylor cone was observed owing to the wetting of the nozzle tip, the stable electrospraying of water without pulsation of the Taylor cone was performed using a pyramidal micronozzle film^25,29^. The localized water meniscus at the pyramidal micronozzle and the electric field concentrated by the in-plane extractor affected the stable formation of the Taylor cone and jet. Consequently, it was confirmed that the operation domain of the pyramidal micronozzle was larger than the domain plotted from the results of a previous study^25^. The pyramidal micronozzle could also be operated by two high-voltage power supply configurations, which were also confirmed to be operated in the same flow rate region (Supplementary Fig. S3). The charged water microdroplets were sprayed without the guide ring, which increased the applicability of the pyramidal micronozzle.

**Fig. 7:**
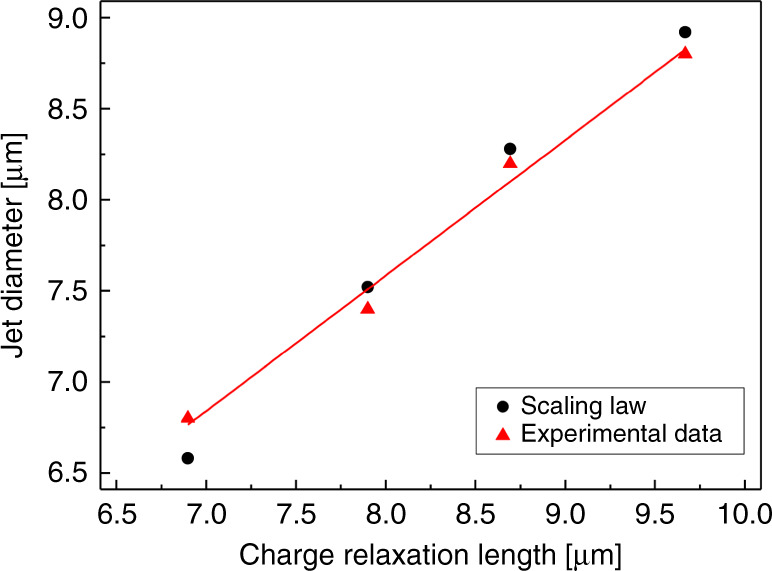
Relationship between the charge-relaxation length and jet diameter. The data plotted by the measurement of the jet diameter showed good agreement with the scaling law for steady cone-jet mode, which was suggested in the classical theory

In the cone-jet mode, the jet radius can be empirically estimated by following the scaling law^15,17,33,34^:

$$r_j = \left( {\frac{{Q\varepsilon _0\kappa ^{1/2}}}{{\pi ^2K}}} \right)^{1/3} = \frac{{\kappa ^{1/2}}}{{\pi ^{2/3}}}r^ \ast ,\;r^ \ast = \left( {\frac{{Q\varepsilon _O\kappa }}{K}} \right)^{1/3}$$

where *r*_*j*_ is the jet radius, and $r^ \ast$ is the charge-relaxation length. Figure 7 shows the relationship between the charge-relaxation length and the jet diameter. The jet diameter was measured using ImageJ software (NIH, Bethesda, MD, USA) based on the images acquired from the DSLR camera. The jet diameter was measured to be in the range of 3.4–4.4 μm. As expected, a linear relationship between the jet diameter and charge-relaxation length was confirmed, which provided further evidence of the electrospraying of water in the steady cone-jet mode.

### Multiplexed electrospraying of water in cone-jet mode

**Fig. 8:**
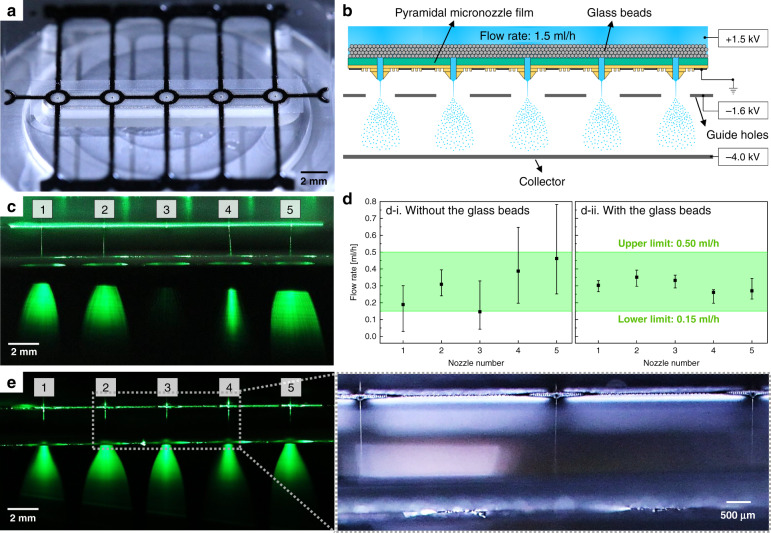
Multiplexed electrospraying of water in cone-jet mode using a pyramidal micronozzle film. **a** Linear array of the five pyramidal micronozzles. The film was also fabricated based on the UV-embossing process, which is demonstrated in Fig. 3b. **b** Schematic setup for the multiplexed electrospraying of water. **c** Visualization of the multiplexed electrospraying of water without the glass beads. Nonuniform multiplexed electrospraying was visualized. **d** Effect of flow homogenization by the glass beads. Flow redistribution by the glass beads reduced the inequality of the flow rates. **e** Visualization of the multiplexed electrospraying of water with the glass beads. All of the nozzles were operated in cone-jet mode

The multiplexed electrospraying of water in cone-jet mode using the pyramidal micronozzle film with five nozzles is shown in Fig. 8. Because the diameter of the pendant drop was 1.4 mm (Supplementary Fig. S1b), the spacing of the micronozzles was set to 4 mm (Fig. 8a). This spacing prevented the coalescence of droplets from the adjacent nozzles when operating at a low voltage. The spacing could be reduced if the voltage for the cone-jet mode was applied before the start of the water flow. The flow rate was set to 1.5 ml/h (0.3 ml/h per nozzle). Four to six layers of glass beads 0.8 mm in diameter were placed upstream of the nozzles to ensure homogenization of the flow (Fig. 8b). The syringe pump stalled when the number of layers exceeded eight because of the high pressure inside the water reservoir. The glass beads were cleaned using ethanol and dried in a vacuum oven before being placed upstream of the nozzle. When multiplexed electrospraying of water was conducted without glass beads, uniform generation of water microdroplets was difficult to achieve (Fig. 8c). In most cases, a weak sprayed water flow was detected on nozzle number 3, whereas nozzle numbers 4 and 5 experienced excessive supplies of incoming water. These nozzles were shown to operate outside the bounds of the cone-jet mode (0.15–0.50 ml/h). One of the main reasons for the unequal flow is the mismatch in surface tension at the solid/water interface^37^. Because the critical surface tension of polycarbonate (~0.042 N/m) is lower than the surface tension of water (~0.072 N/m), it is difficult for water to adhere to the film. This may generate air bubbles and block the flow path to the inlets of the micronozzles^37–39^. The flow homogenization was experimentally demonstrated by observing the pendant drops of the nozzles without applied voltages (Fig. 8d). The volume of the water droplets by pendant drop was measured to be 1.34 μl using ImageJ software. The number of drops at each nozzle was counted for 10 min. Counting was performed in triplicate, and the flow rate was estimated by dividing the total volume of water droplets by the time of observation. As expected, a severe deviation in the flow rate occurred when there were no glass beads upstream of the nozzles (Fig. 8d-i). The flow rates of most of the nozzles were outside the bounds of the cone-jet mode, which led to the nonuniform multiplexed electrospray, as shown in Fig. 8c. The flow rate at each nozzle was measured to be inside the bounds of the cone-jet mode when the glass beads were placed upstream of the nozzles (Fig. 8d-ii). The diversification of stream paths by the three-dimensional pores inside the glass beads allowed the redistribution of water, which prevented severe inequality in the water distribution to the nozzles. The glass beads also reduced the number of air bubbles upstream of the nozzles owing to their high critical surface tension (~0.1 N/m)^40^. Although the effect of the electric capillary force by the applied voltage was not reflected in this experimental flow rate estimation, flow homogenization by the glass beads was identified. Uniform multiplexed electrospraying of water in cone-jet mode was performed using glass beads (Fig. 8e). The voltage was set to 1.5 kV, which is slightly higher than the onset voltage measured in the electrospray of water using a single pyramidal micronozzle at a flow rate of 0.3 ml/h. All five pyramidal micronozzles were observed to operate in cone-jet mode without electrical discharge and intermittence. The operating range of the cone-jet mode for the multiplexed electrospray of water was measured to be 1.5–1.9 kV. The reduced operation domain of the multiplexed electrospray was due to the slight deviation in the flow rates at each nozzle. The multiplexed electrospray of water did not operate when the in-plane extractor was not used due to the electric field crosstalk between neighboring micronozzles, as demonstrated in Supplementary Fig. S4. This result indicates that the in-plane extractor effectively reduced the crosstalk by localizing the electric field at the nozzle tip. The possibility of the uniform multiplexed electrospraying of water at an increased flow rate of 2.0 ml/h was reduced because some of the nozzles operated at flow rates outside the bounds of cone-jet mode. This operation is the remaining task for enlarging the operation domain of the multiplexed electrospray in cone-jet mode. However, there is potential to increase the throughput of the electrospray by using a two-dimensional array of pyramidal micronozzles, which is demonstrated in Supplementary Fig. S5.

In summary, multiplexed electrospraying of water in cone-jet mode was demonstrated using a UV-embossed pyramidal micronozzle film. The pyramidal structure of the nozzle enabled the sharpening of the nozzle tip, which is essential for avoiding unstable electrospray caused by the wetting of the nozzle. The pyramidal structure of the nozzle was also suitable for the UV-embossing process, which was rapid and reproducible. The in-plane extractor was patterned in a precise alignment with the pyramidal micronozzle, allowing the electric field to be concentrated at the tip of the nozzle. The electrospray with the pyramidal micronozzle film was observed using high-speed imaging. The dripping, spindle, and cone-jet modes were categorized depending on the voltage and spray behavior. The onset and end voltages were also measured to identify the cone-jet mode region. It was confirmed that the pyramidal micronozzle had a larger operation domain than the domain represented in a previous study. The multiplexed electrospraying of water in cone-jet mode was operated at a flow rate of 1.5 ml/h with a pyramidal micronozzle film in a single-input multioutput (SIMO) flow system. Flow homogenization effects were also demonstrated. This result shows that the proposed pyramidal micronozzle film has a high potential to overcome the low-throughput characteristics of electrospraying and the high surface tension of water.

## Materials and methods

A 4-inch single-crystal silicon substrate (orientation: (1 0 0), thickness: 525 μm) was used for the fabrication of the mold. The photoresists AZ5214E (Clariant Corp, Switzerland) and THB-151N (JSR Corp, Japan) were used for the patterning of the mold. These photoresists were removed with acetone (Daejung Chemicals, Republic of Korea) and STR-F (JSR Corp, Japan) solution, respectively. The silicon nitride was etched for 4 min using a reactive ion etching process at an applied power of 50 W (RIE machine, Ultech Corp, Republic of Korea). CF_4_ and O_2_ were used for the reactive ion etching process at flow rates of 16 sccm and 4 sccm, respectively. Chromium and copper layers were deposited using an electron beam evaporation machine (EB 500 series, Alpha-plus, Republic of Korea). After electroplating the nickel master mold, the chromium and copper layers were removed using Cr etch 473 solution (Transene Inc, USA) and Cu etch 49-1 solution (Transene Inc, USA), respectively. The pyramidal micronozzle film was embossed using a UV curing system (Sejong Technology, Republic of Korea). A polycarbonate film was drilled using a femtosecond laser machining system (SM-LMM-9100, SM Tech, Republic of Korea).

A scanning electron microscope (SEM, SU5000, Hitachi, Japan) was used to observe the surface morphology of the pyramidal micronozzle film. The surface tension and contact angle of water were measured using a goniometer (DSA10, Krüss, Germany). The electrical conductivity of water was measured by a conductivity meter (CM-31P, DKK Toa Corp, Japan).

High-voltage power supplies (±30 kV, Korea Switching, Republic of Korea) and a syringe pump (KDS 100, KD Scientific, USA) were used for the electrospray operation. The applied voltages were measured using a multimeter (Model 189, Fluke, USA) with a high-voltage probe. The electrospraying of water was visualized by a DSLR camera (Canon 80D, Japan) and a high-speed camera (FASTCAM Mini AX200, Photron, USA).

## Supplementary information

Revised supplementary material - marked up

Revised supplementary material - clean version
