# Supplementary material for: Multiplexed electrospraying of water in cone-jet mode using a UV-embossed pyramidal micronozzle film
Source: Microsyst Nanoeng. 2022 Sep 29;8:110. doi: 10.1038/s41378-022-00391-1 (PMC9522652; doi:10.1038/s41378-022-00391-1)
Supplement: Supplementary file 2 — Revised supplementary material - clean version [file 41378_2022_391_MOESM2_ESM.docx]

**Supplementary material**

**
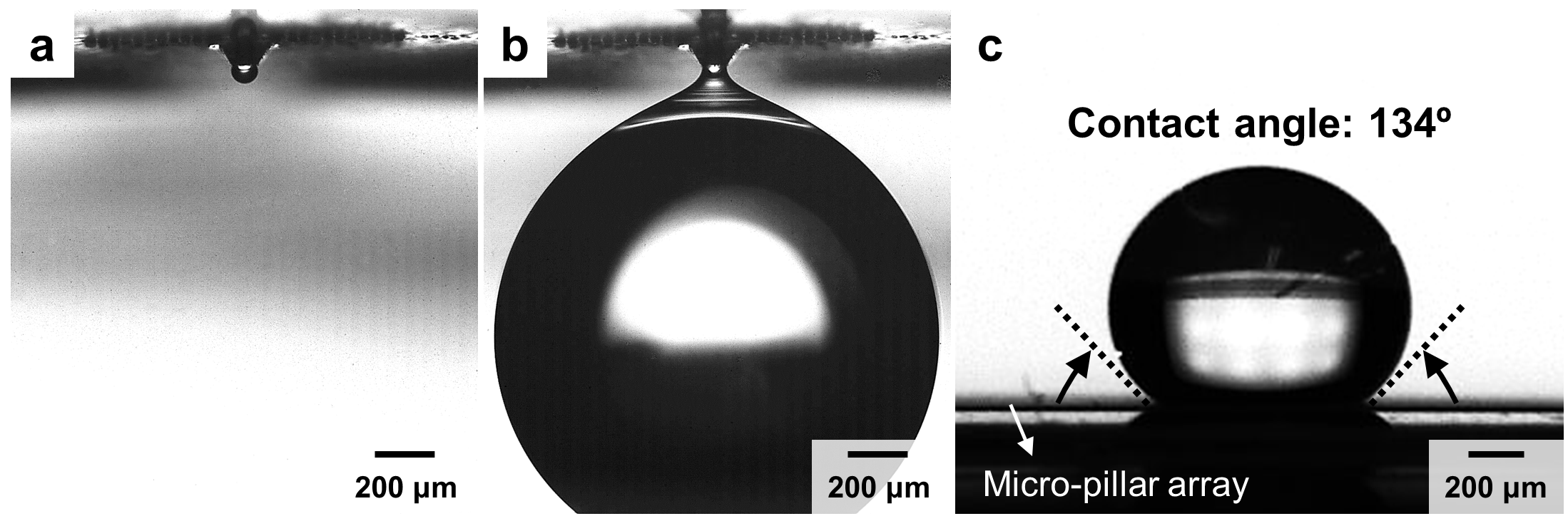
**

**Fig. S1.** Images of pendant drop from the pyramidal micro-nozzle and contact angle measurement.

**Fig. S1a.** Initial state (0 ms).

**Fig. S1b.** Final state (2983 ms, The state just before the droplet falls.)

Fig. S1a and Fig. S1b show images of the pendant drop from the pyramidal micro-nozzle. The images were recorded at a time interval of 1 ms (1000 fps) and an exposure time of 260 ns. The flow rate was set at 0.1 ml/h. The water meniscus anchored at the inner rim of the nozzle during the pendant drop test. The static contact angle of the micro-pillar array was 134° in Cassie Baxter state (**Fig. S1(c)**)


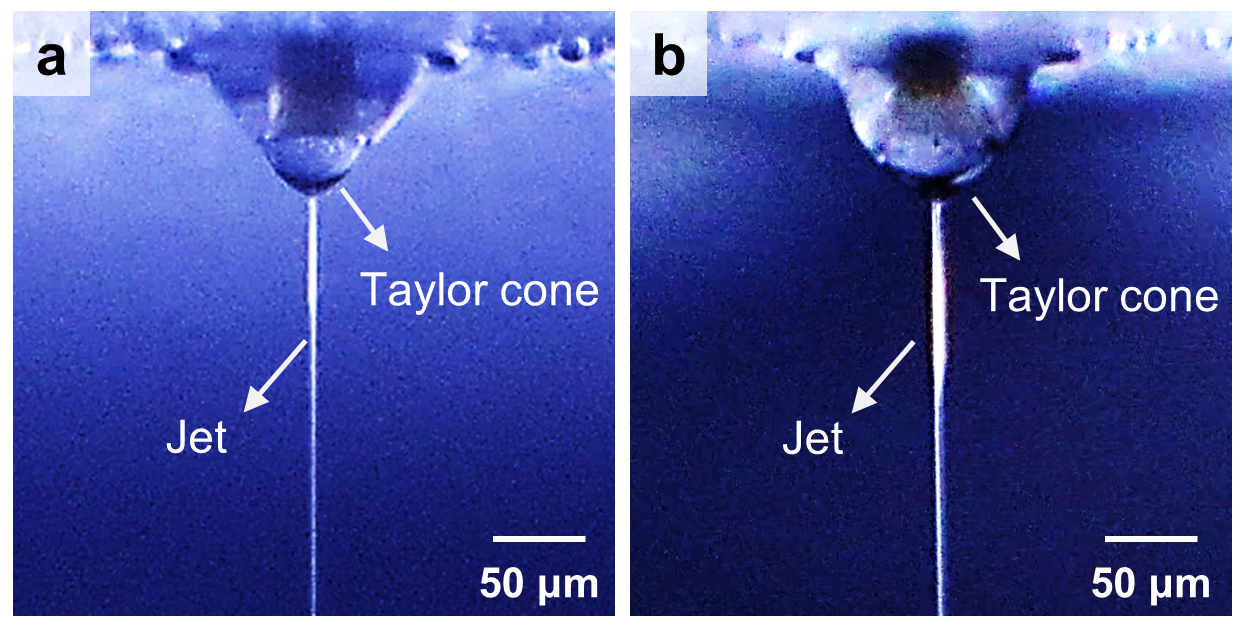


**Fig. S2.** Electrospray of water in the cone jet mode depending on the wetting status of the pyramidal micro-nozzle tip (Voltage at water: 1.6 kV, Flow rate: 0.50 ml/h)

**Fig. S2a.** Electrospray of water in the cone jet mode when the pyramidal nozzle tip is hydrophobic.

**Fig. S2b.** Electrospray of water in the cone jet mode when the pyramidal nozzle tip is hydrophilic. Even though the nozzle tip was wet, steady cone jet mode was still observed.


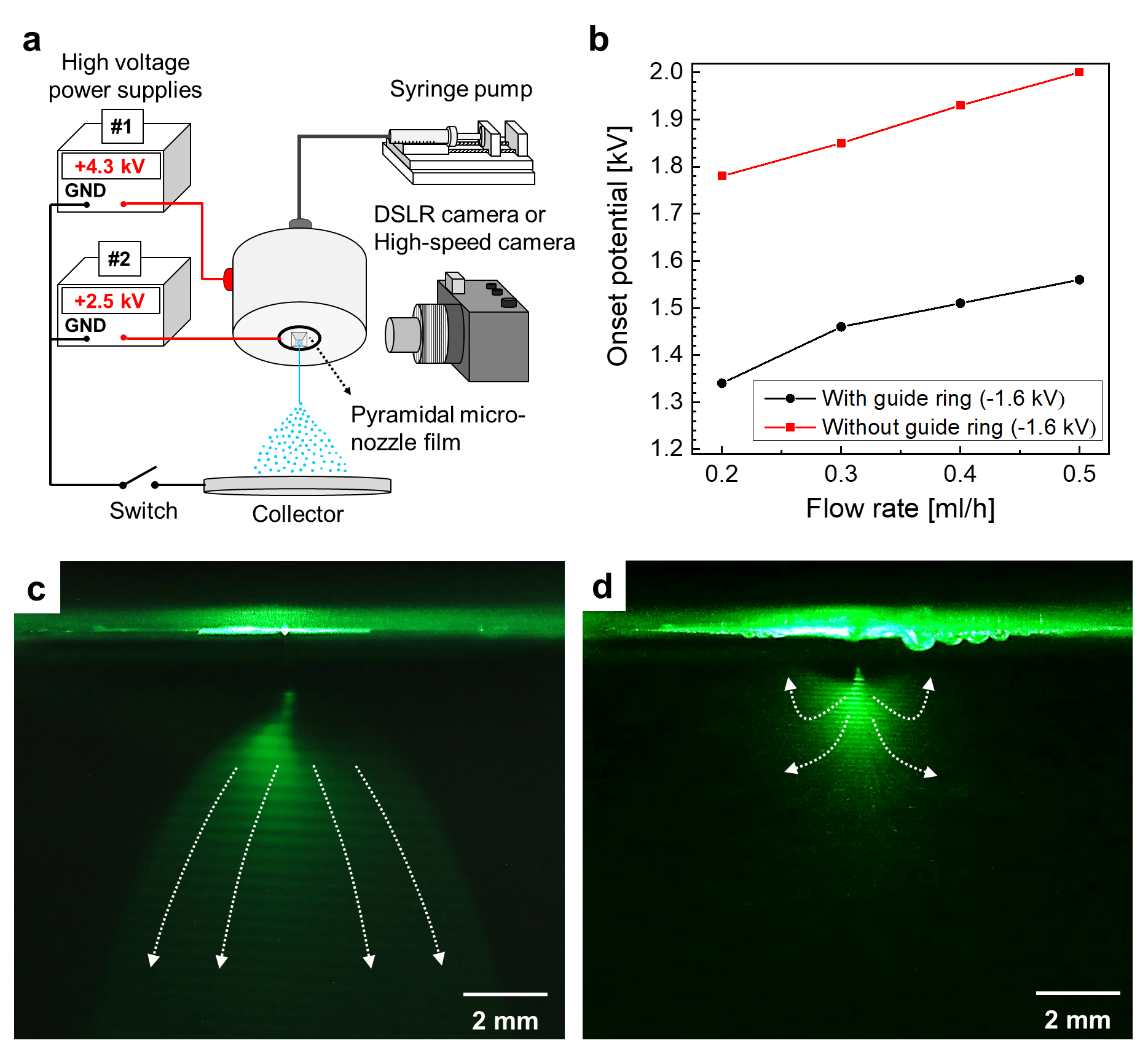


**Fig. S3.** Electrospray by two high voltage power supplies configuration

**Fig. S3a.** Experiment setup of two high voltage power supplies configuration. The voltage at the in-plane extractor of the pyramidal micro-nozzle film was fixed to +2.5 kV.

**Fig. S3b.** Onset potential difference between the setup in Fig. 4 (with guide ring) and Fig. S3a (without guide ring). Onset potential is the voltage difference between the water and the in-plane extractor. Due to the negative potential −1.6 kV applied at the guide ring, the onset potential of the two high voltage power supplies setup was higher.

**Fig. S3c.** Electrospray of water in the cone jet mode when the switch to was connected to the grounded collector. The charged water micro-droplets were driven to the grounded collector.

**Fig. S3d.** Electrospray of water in the cone jet mode when the switch was disconnected. The charged water micro-droplets were sprayed randomly. Some of the droplets were accumulated on the in-plane extractor.


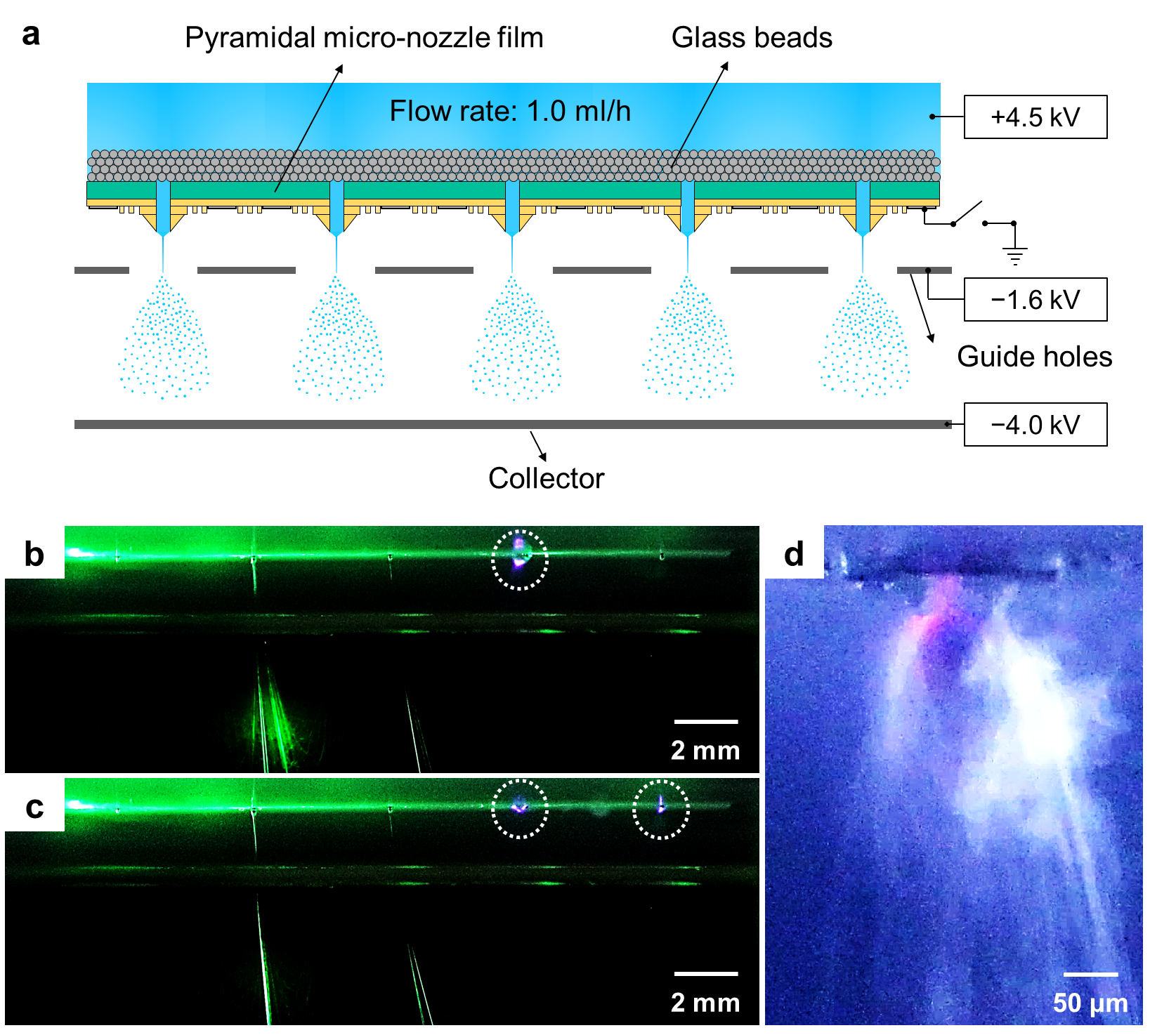


**Fig. S4**. Multiplexed electrospray of water without the in-plane extractor

**Fig. S4a.** Schematics of multiplexed electrospray of water without using the in-plane extractor

**Fig. S4b.** Electrical discharge at the 4^th^ nozzle (0 ms)

**Fig. S4c.** Electrical discharge at the 4^th^ and the 5^th^ nozzle (0.5 s)

**Fig. S4d.** DSLR camera image of electrical discharge at the tip of the pyramidal micro-nozzle.

The circle dotted lines are the nozzles under electrical discharge.


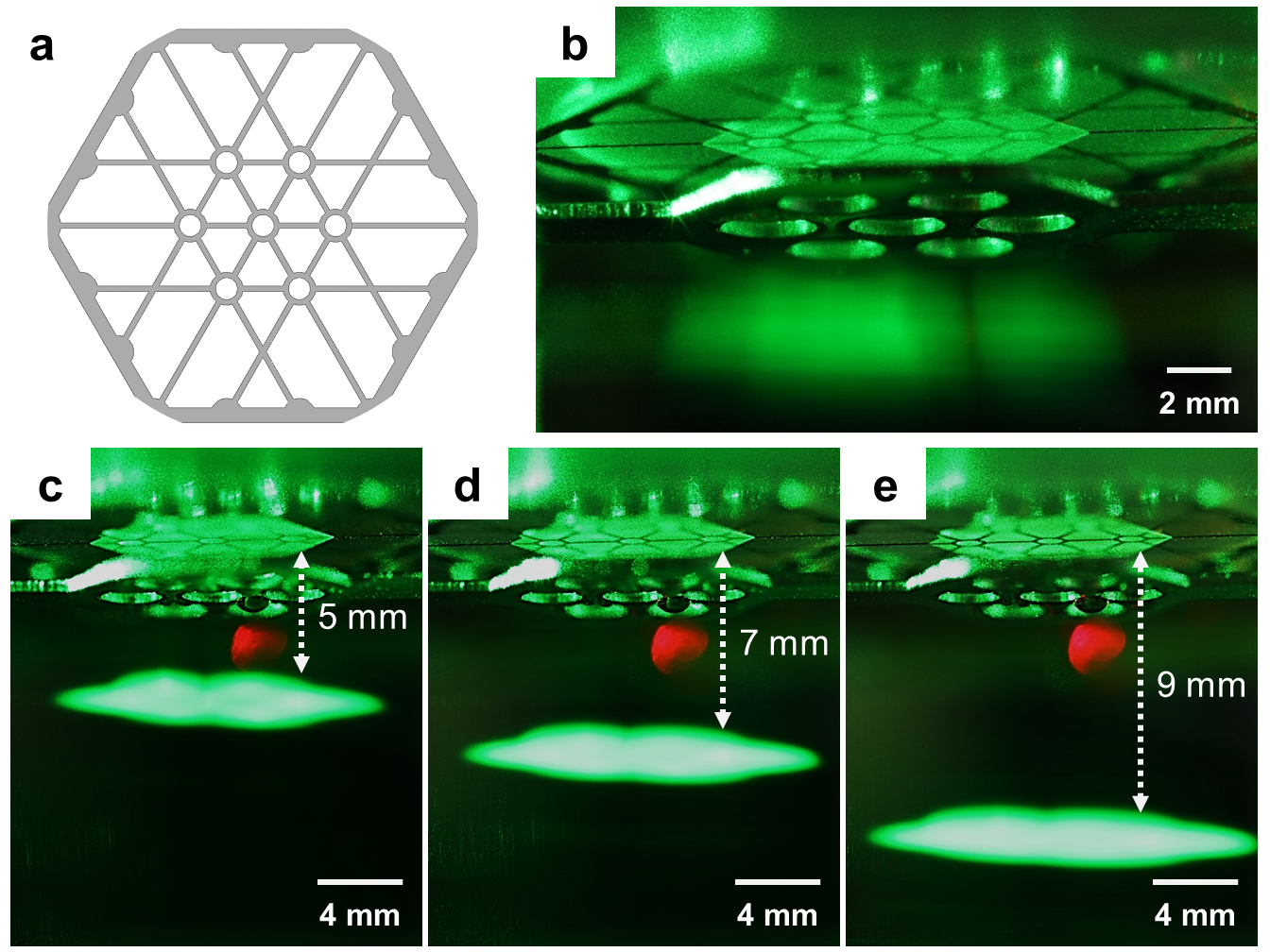


**Fig. S5**. Multiplexed electrospray of water using a 2-dimensional array of seven micro-nozzles.

**Fig. S5a.** In-plane extractor design of the 2-dimensional array in triangular arrangement with the spacing of 4 mm

**Fig. S5b.** Visualization of the 2-dimensional multiplexed electrospray of water. The laser sheet was perpendicular to the in-plane extractor and the view direction of the camera.

**Fig. S5c-e.** Water micro-droplets were visualized by the laser sheets parallel to the in-plane extractor and the view direction of the camera. The laser sheets were placed 5 mm (Fig. S5c), 7 mm (Fig. S5d), and 9 mm (Fig. S5e) away from the film.
